# Association of Systolic Blood Pressure Elevation With Disproportionate Left Ventricular Remodeling in Very Preterm-Born Young Adults: The Preterm Heart and Elevated Blood Pressure

**DOI:** 10.1001/jamacardio.2021.0961

**Published:** 2021-05-12

**Authors:** Afifah Mohamed, Maciej Marciniak, Wilby Williamson, Odaro J. Huckstep, Winok Lapidaire, Angus McCance, Stefan Neubauer, Paul Leeson, Adam J. Lewandowski

**Affiliations:** 1 Oxford Cardiovascular Clinical Research Facility, Division of Cardiovascular Medicine, Radcliffe Department of Medicine, University of Oxford, Oxford, England; 2 Department of Diagnostic Imaging & Applied Health Sciences, Faculty of Health Sciences, Universiti Kebangsaan Malaysia, Kuala Lumpur, Malaysia; 3 Oxford University Hospitals NHS Foundation Trust, Oxford, England; 4 Department of Biomedical Engineering, Division of Imaging Sciences and Biomedical Engineering, King's College London, London, England; 5 Department of Biology, United States Air Force Academy, Air Force Academy, Colorado; 6 Oxford Centre for Clinical Magnetic Resonance Research, Division of Cardiovascular Medicine, Radcliffe Department of Medicine, University of Oxford, Oxford, England

## Abstract

**Importance:**

Preterm-born individuals have higher blood pressure with an increased risk of hypertension by young adulthood, as well as potentially adverse cardiac remodeling even when normotensive. To what extent blood pressure elevation affects left ventricular (LV) structure and function in adults born preterm is currently unknown.

**Objective:**

To investigate whether changes observed in LV structure and function in preterm-born adults make them more susceptible to cardiac remodeling in association with blood pressure elevation.

**Design, Setting, and Participants:**

This cross-sectional cohort study, conducted at the Oxford Cardiovascular Clinical Research Facility and Oxford Centre for Clinical Magnetic Resonance Research, included 468 adults aged 18 to 40 years. Of these, 200 were born preterm (<37 weeks’ gestation) and 268 were born at term (≥37 weeks’ gestation). Cardiac magnetic resonance imaging was used to characterize LV structure and function, with clinical blood pressure readings measured to assess hypertension status. Demographic and anthropometric data, as well as birth history and family medical history information, were collected. Data were analyzed between January 2012 and February 2021.

**Main Outcomes and Measures:**

Cardiac magnetic resonance measures of LV structure and function in response to systolic blood pressure elevation.

**Results:**

The cohort was primarily White (>95%) with a balanced sex distribution (51.5% women and 48.5% men). Preterm-born adults with and without hypertension had higher LV mass index, reduced LV function, and smaller LV volumes compared with term-born individuals both with and without hypertension. In regression analyses of systolic blood pressure with LV mass index and LV mass to end-diastolic volume ratio, there was a leftward shift in the slopes in preterm-born compared with term-born adults. Compared with term-born adults, there was a 2.5-fold greater LV mass index per 1–mm Hg elevation in systolic blood pressure in very and extremely preterm-born adults (<32 weeks’ gestation) (0.394 g/m^2^ vs 0.157 g/m^2^ per 1 mm Hg; *P* < .001) and a 1.6-fold greater LV mass index per 1–mm Hg elevation in systolic blood pressure in moderately preterm-born adults (32 to 36 weeks’ gestation) (0.250 g/m^2^ vs 0.157 g/m^2^ per 1 mm Hg; *P* < .001). The LV mass to end-diastolic volume ratio per 1–mm Hg elevation in systolic blood pressure in the very and extremely preterm-born adults was 3.4-fold greater compared with those born moderately preterm (3.56 × 10^−3^ vs 1.04 × 10^−3^ g/mL per 1 mm Hg; *P* < .001) and 3.3-fold greater compared with those born at term (3.56 × 10^−3^ vs 1.08 × 10^−3^ g/mL per 1 mm Hg; *P* < .001).

**Conclusions and Relevance:**

Preterm-born adults have a unique LV structure and function that worsens with systolic blood pressure elevation. Additional primary prevention strategies specifically targeting cardiovascular risk reduction in this population may be warranted.

## Key Points

**Question:**

Are left ventricular structure and function in preterm-born adults more susceptible to remodeling in association with blood pressure elevation?

**Findings:**

In this cross-sectional cohort study of 468 adults with cardiac magnetic resonance imaging, left ventricular mass index and mass to end-diastolic volume ratio were greater for each 1–mm Hg elevation in systolic blood pressure in preterm-born adults than in term-born adults, with the greatest rise in those born very and extremely preterm (<32 weeks’ gestation).

**Meaning:**

The findings of this study show that adults born preterm demonstrate greater remodeling in response to systolic blood pressure elevation and may require earlier interventions to prevent cardiovascular disease progression.

## Teaser

This study investigates whether changes observed in left ventricular structure and function in preterm-born adults make them more susceptible to cardiac remodeling in association with blood pressure elevation.

## Introduction

Individuals born preterm are at greater relative risk of early heart failure^1^ and ischemic heart disease,^2^ with risk increasing according to the degree of prematurity. Part of this risk may be associated with altered cardiac physiology because preterm-born individuals have potentially adverse structural and functional changes across developmental stages from birth to young adulthood.^3^ This includes impaired left ventricular (LV) diastolic function, smaller internal LV dimensions, and a greater rate of LV hypertrophy from childhood to young adulthood.^3^

In a rat model of preterm birth conditions, early cardiac remodeling, similar to that observed in humans, makes rodents more susceptible to heart failure following hemodynamic pressure overload.^4,5^ In humans, the preterm heart is less able to cope under acute physiological stress, with impairments in ejection fraction, stroke volume, and cardiac output during moderate- to high-intensity exercise.^6,7^ It is thus conceivable that the observed patterns of cardiac remodeling seen in individuals born preterm make their hearts more vulnerable to chronic insults, such as the hemodynamic stress of sustained blood pressure elevation. Of related concern is that, compared with their term-born peers, individuals born preterm have higher systolic and diastolic blood pressures,^8^ with preterm birth now recognized as a risk factor for hypertension in adulthood.^9^

While earlier studies have shown associations between cardiac structural and functional alterations with increasing blood pressure,^10,11^ they have been limited by small numbers of individuals with blood pressures in the clinically elevated and hypertensive range. We have therefore performed a cardiac magnetic resonance (CMR) imaging study in 468 individuals, of whom more than 25% were clinically hypertensive, to determine how systolic blood pressure elevation may be associated with LV structure and function in adults born preterm. Furthermore, we have investigated whether the strength of the association between LV changes and systolic blood pressure is dependent on the degree of prematurity. We hypothesized that preterm-born individuals have an altered cardiac phenotype that is more susceptible to further cardiac remodeling in association with blood pressure elevation.

## Methods

### Study Population

Participants were recruited as part of a program of ongoing research in Oxford, England, into the association between preterm birth and cardiovascular outcomes. All participants had been identified through open recruitment including prospective recruitment from birth, targeted online recruitment, as well as invitation from general practice records, the local hypertension clinic, and hospital birth registers. Inclusion and exclusion criteria for this analysis included being between ages 18 and 40 years, having a body mass index (BMI; calculated as weight in kilograms divided by height in meters squared) less than 40, and no history of hypertension treatment, cardiac, or cerebrovascular disease. All research study visits were conducted at the Oxford Cardiovascular Clinical Research Facility (CCRF) and Oxford Centre for Clinical Magnetic Resonance Research (OCMR). Birth and family history data were attained via a combination of prospective collection, access to medical notes, and by questionnaire. Individuals were classified based on gestational age as either being born preterm (<37 weeks’ gestation) or at term (≥37 weeks’ gestation). Classification of normotensive and hypertensive was based on cutoff levels derived from the American College of Cardiology/American Heart Association blood pressure classification guidelines (systolic blood pressure ≥130 mm Hg and/or diastolic blood pressure ≥80 mm Hg).^12^ Data were coded with participant- and study-specific identifications to ensure anonymity and blinded analysis. Ethical approval was granted by the South Central Oxford A research ethics committee, the South Central Berkshire research ethics committee, and the South Central Oxford B research ethics committee. All participants provided signed informed consent.

### Study Visit

#### Anthropometry, Blood Samples, and Blood Pressure Measurements

All participants fasted for a minimum of 6 hours before their study visit. As previously described,^7,10,11,13^ trained clinical research investigators completed all height and weight measurements as well as blood sample collection. Participants were seated for 5 minutes before 3 resting brachial blood pressure readings (Dinamap V100; GE Healthcare) were taken on the left arm with a 1-minute interval in between. These measurements were done on the same day as the CMR scan. The final 2 readings were averaged for analysis. Twenty-four–hour ambulatory blood pressure monitoring and central blood pressure measurements were done in a subgroup as part of the same study visit (eMethods in the Supplement).

#### Cardiac Magnetic Resonance Acquisition

Cardiac magnetic resonance imaging was performed on a 1.5-T or 3.0-T magnetic resonance scanner (1.5-T Sonata and 3-T TIM Trio; Siemens Medical Solutions) to quantify LV structure and function. A previous comparison performed by our center showed the feasibility and consistency of cardiac volume and mass measurements across field strengths.^14^ Horizontal and vertical long-axis retrospective electrocardiogram-gated steady-state free precession cine images were acquired, followed by short-axis steady-state free precession cine images.^10^ Data were acquired at end-expiratory breath-hold and digitally stored for offline analysis.

### Image Analysis

#### Quantification of LV Volumes, Mass, Dimensions, and Function

All LV analysis was done using analytic software (CVI42; Circle Cardiovascular Imaging). To evaluate LV volumes, mass, and dimensions, both the epicardial and endocardial borders were manually contoured on short-axis cine images for each slice at end diastole and endocardial borders at end systole. The end-diastolic and end-systolic cardiac phases and basal and apical LV slices were visually determined as previously described.^10,15^ Average LV wall thickness was measured on the midventricular short-axis slice at end diastole. Relative wall thickness (RWT) was calculated on the midventricular short-axis slice at end diastole as the LV inferolateral plus anteroseptal wall thickness, all divided by LV cavity diameter. Length of the LV was measured as the length from the LV apex to the middle of the mitral valve annulus at end diastole. Sphericity index was calculated as the ratio of LV end-diastolic volume, divided by the volume of a sphere, with LV length as the diameter. Myocardial deformation analysis was performed using feature tracking. Endocardial and epicardial borders of the LV for the horizontal long-axis cine and basal, mid, and apical short-axis cines were manually contoured on the end-diastolic frame. The deformation of the myocardium was then automatically tracked through the phases of the cardiac cycle.

### Statistical Analysis

Statistical analysis was performed using SPSS, version 25 (IBM) and R, version 3.6.1 (R Foundation for Statistical Computing). All statistical analyses were done based on a comparison of birth history (preterm and term groups) and gestational age categories (grouped as <32 weeks, 28-31 weeks, 32-36 weeks, and ≥37 weeks). Data were presented as mean (SD) or number (percentage) for all comparisons. Shapiro-Wilk testing was used to verify data normality. To compare continuous variables between groups, we used the *t* test for normally distributed data and the Mann-Whitney test for skewed data. All group comparisons of birth history and gestational age categories were adjusted for age and sex. After bivariate regression analyses, multivariable regression analyses were conducted to identify the association between key LV parameters (dependent variables) and systolic blood pressure, adjusting for potential confounders (age, sex, birth weight *z* score, and BMI). For each analysis, unstandardized coefficients (B) with 95% confidence intervals and *P* values were computed. One-way analysis of covariance (ANCOVA) was conducted to determine whether there were significant differences in the regression lines between the groups. Sum squares, *F* statistics, and *P* values were calculated for each parameter within each model. *P* values of .05 or less were considered statistically significant unless multiple subgroup comparisons were made, in which case, 2-tailed *P* values of .01 or less were considered statistically significant.

## Results

### Baseline Participant Characteristics

A total of 493 participants recruited to the Oxford program on preterm birth and cardiovascular outcomes had undergone CMR scans. For this study, 25 participants were then excluded: 3 owing to missing clinic blood pressure readings; 16 who were receiving antihypertensive medications; 4 who had a BMI of at least 40; and 2 who had preexisting cardiac conditions. Of the remaining 468 participants, 200 were born preterm and 268 were born at term. There were no significant differences in age and sex between the preterm-born and term-born groups (eTable 1 in the Supplement). Of 200 individuals born preterm, 52.5% (n = 105) were born moderately preterm (32-36 weeks’ gestation), 37.0% (n = 74) were born very preterm (28-31 weeks’ gestation), and 10.5% (n = 21) were born extremely preterm (<28 weeks’ gestation). The preterm-born individuals were shorter and weighed less than those born at term, but there was no difference in BMI between groups. Only a small percentage of preterm-born and term-born adults were born small for gestational age (5.0% and 1.9%, respectively). When dividing birth history groups by blood pressure status, within both the preterm-born and term-born groups, there were more male individuals among the individuals with hypertension, who were also slightly older compared with their peers without hypertension (Table 1). A family history of hypertension was also more prevalent in preterm-born adults with hypertension (eTable 2 in the Supplement) so additional analyses adjusting for family history of hypertension are provided in the Supplement. There were similar magnitudes of systolic and diastolic blood pressure differences between the normotensive and hypertensive subgroups within the preterm-born individuals (systolic: +15.1%; *P* < .001; diastolic: +16.4%, *P* < .001) and term-born individuals (systolic: +15.7%; *P* < .001; diastolic: +20.3%; *P* < .001).

**Table 1.** Baseline Participant Characteristics Divided by Birth History and Blood Pressure Subgroups

Characteristic	Preterm-born adults, mean (SD)		*P* value^a^	Term-born adults, mean (SD)		*P* value^b^	*P* value^c^	*P* value^d^	*P* value^e^
	NT (n = 139)	HT (n = 61)		NT (n = 205)	HT (n = 63)				
Demographics and anthropometrics									
Age, y	25.4 (3.95)	26.4 (3.84)	.12	26.1 (4.41)	27.8 (4.93)	.01	.13	.001	.10
Male, No. (%)	56 (40.3)	35 (57.4)	.03	94 (45.9)	42 (66.7)	.005	.32	.002	.35
Female, No. (%)	83 (59.7)	26 (42.6)		111 (54.1)	21 (33.3)				
Height, cm	169 (9.69)	172 (10.85)	.62	173 (9.24)	176 (8.60)	.53	<.001	.001	.01
Weight, kg	67.1 (13.09)	74.1 (13.11)	.01	70.4 (12.41)	78.7 (13.04)	.002	.05	<.001	.14
BMI,	23.5 (3.62)	25.1 (3.75)	.008	23.5 (3.23)	25.4 (3.55)	.002	.69	.009	.97
Birth weight, g	1600 (606)	1692 (687)	.39	3429 (410)	3499 (464)	.23	<.001	<.001	<.001
Birth weight, *z* score	−0.23 (1.01)	−0.34 (1.14)	.44	0.10 (0.84)	0.05 (1.04)	.72	.001	.05	.04
Gestational age, wk	31.17 (2.99)	31.77 (2.99)	.17	39.50 (1.11)	39.86 (1.20)	.05	<.001	<.001	<.001
<28 wk, No. (%)	14 (10.1)	7 (11.5)	.67	NA	NA	NA	NA	NA	NA
28-31 wk, No. (%)	55 (39.6)	19 (31.1)	.13	NA	NA	NA	NA	NA	NA
32-36 wk, No. (%)	70 (50.4)	35 (57.4)	.23	NA	NA	NA	NA	NA	NA
Biochemistry									
Total cholesterol, mg/dL	182.2 (36.7)	174.9 (46.3)	.30	170.3 (35.5)	177.6 (40.9)	.39	.001	.56	.74
HDL, mg/dL	59.1 (14.3)	54.1 (13.5)	.13	56.4 (13.5)	52.5 (12.4)	.53	.13	.13	.97
LDL, mg/dL	107.3 (27.8)	106.6 (35.9)	.67	97.7 (28.2)	108.9 (35.5)	.12	<.001	.96	.93
Triglycerides, mg/dL	44.0 (33.6)	54.8 (43.6)	.13	36.3 (22.0)	44.8 (24.7)	.09	.005	.85	.06
High-sensitivity CRP, mg/dL	0.21 (0.40)	0.16 (0.25)	.38	0.15 (0.33)	0.19 (0.29)	.39	.15	.54	.43
Glucose, mmol/L	88.5 (2.70)	91.2 (6.67)	.07	85.6 (8.11)	91.0 (7.93)	.001	.001	.36	.41
Insulin, uIU/mL	7.62 (4.64)	7.92 (3.97)	.32	5.92 (4.24)	9.70 (10.67)	.001	.001	.07	.34
HOMA-B (%)	99.1 (34.92)	95.6 (31.95)	.79	91.4 (29.51)	99.7 (40.38)	.15	.10	.38	.59
HOMA-S (%)	128.0 (56.35)	115.9 (44.86)	.05	166 (71.83)	124 (64.34)	.001	<.001	.41	.37
HOMA-IR	0.82 (0.33)	0.88 (0.32)	.14	0.71 (0.34)	0.94 (0.44)	.002	.01	.07	.67
Brachial blood pressure, mm Hg									
Resting systolic	115.6 (7.33)	133.0 (9.99)	NA	113.7 (8.15)	131.6 (8.50)	NA	NA	NA	NA
Resting diastolic	69.4 (5.43)	80.8 (7.57)	NA	68.5 (6.04)	82.4 (7.36)	NA	NA	NA	NA

Abbreviations: BMI, body mass index (calculated as weight in kilograms divided by height in meters squared); CRP, C-reactive protein; HDL, high-density lipoprotein; HOMA, homeostatic model assessment; HT, hypertensive; LDL, low-density lipoprotein; NA, not applicable; NT, normotensive.

^a^ Preterm-born NT vs preterm-born HT.

^b^ Term-born NT vs term-born HT.

^c^ Preterm-born NT vs term-born NT.

^d^ Preterm-born NT vs term-born HT.

^e^ Preterm-born HT vs term-born HT.

### Left Ventricular Remodeling in Preterm-Born and Term-Born Young Adults With Hypertension

In preterm-born adults, myocardial mass was significantly higher in the hypertensive group compared with the normotensive group (Table 2). This difference in myocardial mass was not significant when indexed to body surface area (Table 2). Other measures of LV structure and function did not differ significantly between blood pressure groups. Within the term-born group, LV ejection fraction was higher in the hypertensive group owing to a lower end-systolic volume. This potential increase in systolic function was supported by greater LV mid-ventricular peak systolic circumferential strain.

**Table 2.** Left Ventricular Structural and Functional Measures Divided by Birth History and Blood Pressure Subgroups

Variable	Preterm-born adults, mean (SD)		*P* value^a^	Term-born adults, mean (SD)		*P* value^b^	*P* value^c^	*P* value^d^	*P* value^e^
	NT (n = 139)	HT (n = 61)		NT (n = 205)	HT (n = 63)				
Volumes and dimensions									
ED volume, mL	128.3 (26.13)	139.3 (31.63)	.09	149.6 (30.64)	156.3 (29.47)	.76	<.001	<.001	.001
ES volume, mL	47.72 (12.29)	51.26 (15.27)	.36	53.99 (14.74)	53.04 (15.33)	.09	<.001	.17	.58
Stroke volume, mL	80.57 (17.31)	87.91 (21.03)	.07	95.09 (20.61)	103.2 (18.47)	.06	<.001	<.001	<.001
ED volume/BSA, mL/m^2^	72.28 (10.28)	73.95 (11.44)	.62	81.15 (12.43)	80.06 (12.92)	.23	<.001	<.001	.005
ES volume/BSA, mL/m^2^	26.82 (5.25)	27.41 (7.00)	.87	29.41 (7.09)	27.20 (7.37)	.005	<.001	.85	.88
Stroke volume/BSA, mL/m^2^	45.46 (7.50)	46.75 (7.91)	.47	51.88 (8.29)	52.85 (7.79)	.67	<.001	<.001	<.001
Length, mm	90.08 (7.01)	93.19 (7.37)	.07	98.76 (7.82)	101.0 (7.91)	.44	<.001	<.001	<.001
Average wall thickness, mm	8.63 (1.32)	9.12 (1.23)	.06	6.96 (1.32)	7.49 (1.10)	.24	<.001	<.001	<.001
Relative wall thickness	0.35 (0.06)	0.36 (0.06)	.39	0.26 (0.05)	0.27 (0.05)	.37	<.001	<.001	<.001
Sphericity index	0.34 (0.06)	0.33 (0.06)	.66	0.30 (0.05)	0.29 (0.06)	.84	<.001	<.001	.001
ED diameter, cm	50.34 (4.98)	51.49 (5.17)	.64	55.0 (4.78)	55.77 (4.62)	.93	<.001	<.001	<.001
Myocardium mass, g	112.5 (26.10)	125.9 (26.26)	.01	101.3 (23.46)	113.6 (23.16)	.03	<.001	.02	<.001
Myocardium mass/BSA, g/m^2^	63.31 (10.41)	67.11 (9.67)	.07	54.81 (9.67)	58.04 (9.60)	.32	<.001	<.001	<.001
Mass/ED volume, g/mL	0.88 (0.12)	0.91 (0.12)	.18	0.68 (0.12)	0.73 (0.11)	.05	<.001	<.001	<.001
Cardiac index, L/min/m^2^	3.20 (0.60)	3.34 (0.67)	.13	3.39 (0.67)	3.51 (0.62)	.23	.006	.003	.12
Heart rate, bpm	70.94 (10.54)	72.07 (10.72)	.22	65.98 (10.07)	66.95 (8.85)	.41	<.001	.07	.008
RWT >0.42, No. (%)	18 (12.9)	11 (18.0)	.54	0	1 (1.6)	.11	<.001	.001	.003
Function									
Ejection fraction, %	62.92 (5.06)	63.29 (6.73)	.56	64.06 (5.15)	66.38 (5.43)	.001	.04	<.001	.007
Longitudinal strain									
Strain, %	−15.14 (2.46)	−15.17 (2.61)	.97	−18.64 (3.53)	−18.92 (3.04)	.29	<.001	<.001	<.001
Strain rate, 1/s	−0.94 (0.27)	−0.97 (0.22)	.39	−1.00 (0.31)	−1.00 (0.27)	.70	.06	.14	.63
Circumferential strain									
Basal strain, %	−19.22 (3.54)	−18.39 (3.94)	.35	−20.02 (3.60)	−20.12 (2.77)	.78	.03	.03	.004
Basal strain rate, 1/s	−1.17 (0.27)	−1.12 (0.24)	.38	−1.14 (0.33)	−1.11 (0.26)	.68	.45	.40	.95
Mid strain, %	−17.75 (2.19)	−17.53 (2.27)	.81	−18.35 (2.23)	−19.34 (2.23)	.005	.008	<.001	<.001
Mid strain rate, 1/s	−1.05 (0.14)	−1.05 (0.12)	.92	−1.04 (0.20)	−1.04 (0.17)	.83	.51	.76	.96
Apical strain, %	−20.23 (3.56)	−19.97 (4.37)	.86	−22.35 (3.38)	−22.78 (3.25)	.27	<.001	<.001	<.001
Apical strain rate, 1/s	−1.42 (0.34)	−1.47 (0.38)	.36	−1.49 (0.45)	−1.53 (0.39)	.38	.07	.03	.38

Abbreviations: BSA, body surface area; ED, end-diastolic volume; ES, end-systolic volume; HT, hypertensive; NT, normotensive; RWT, relative wall thickness.

^a^ Preterm-born NT vs preterm-born HT.

^b^ Term-born NT vs term-born HT.

^c^ Preterm-born NT vs term-born NT.

^d^ Preterm-born NT vs term-born HT.

^e^ Preterm-born HT vs term-born HT.

We compared LV structural and functional measures in preterm-born individuals with term-born individuals with and without hypertension (Table 2; eFigure 1 in the Supplement). Preterm-born individuals without hypertension had smaller LV dimensions and volumes compared with both term-born individuals with and without hypertension, with higher LV mass indexed to body surface area compared with both groups. Although ejection fraction did not differ between preterm-born and term-born normotensive groups, it was significantly higher in the hypertensive term group compared with the preterm-born normotensive group. Longitudinal peak systolic strain as well as midventricular and apical peak systolic circumferential strain measures were lower in the preterm-born normotensive group compared with term-born individuals with and without hypertension. In addition, basal peak systolic circumferential strain was lower in the preterm-born hypertensive group compared with the term-born hypertensive group.

### Association Between Systolic Blood Pressure and LV Parameters

**Figure 1.**
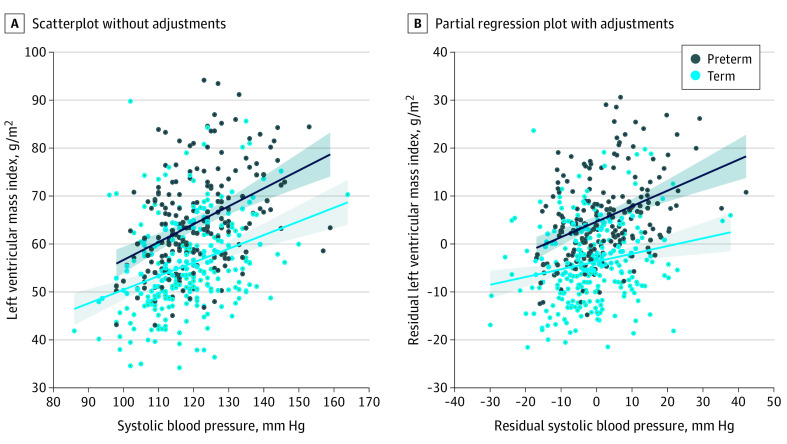
Association Between Systolic Blood Pressure and Left Ventricular (LV) Mass Index in Preterm-Born and Term-Born Adults A, Scatterplot for preterm-born (*R*^2^ = 16.3%; *P* < .001) and term-born (*R*^2^ = 11.0%; *P* < .001) adults. B, Partial regression plot with adjustment for age, sex, birth weight *z* score, and body mass index demonstrates a stronger association in preterm-born than term-born adults (*R*^2^ = 14.6%; *P* < .001 vs *R*^2^ = 3.72%; *P* = .002).

**Figure 2.**
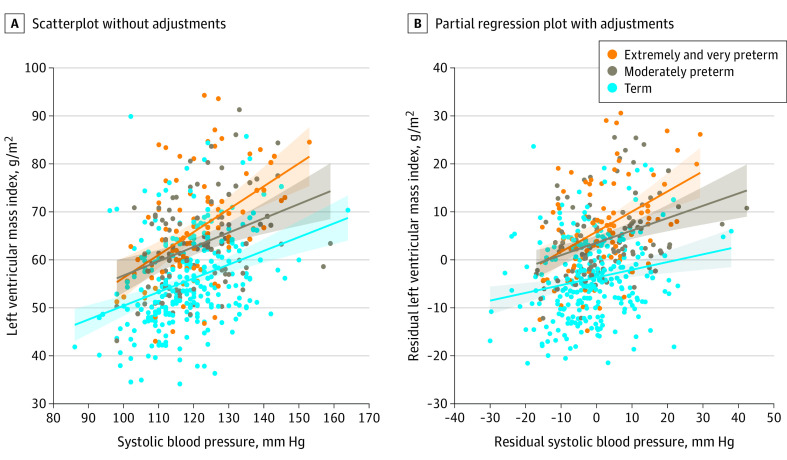
Association Between Systolic Blood Pressure and Left Ventricular (LV) Mass Index Based on Gestational Age Categories A, Scatterplot for extremely/very preterm (*R*^2^ = 23.1%; *P* < .001); moderately preterm (*R*^2^ = 13.6%; *P* < .001); and term (*R*^2^ = 13.6%; *P* < .001). B, Partial regression plot adjusting for age, sex, birth weight *z* score and body mass index. Extremely/very preterm: *R*^2^ = 17.5%; *P* < .001; moderately preterm: *R*^2^ = 12.7%; *P* < .001; term: *R*^2^ = 3.72%, *P* = .002.

The association between systolic blood pressure and LV mass index was significant in both the preterm-born and term-born groups but was greater in those born preterm in the multivariable regression models per 1–mm Hg elevation in systolic blood pressure (0.318 g/m^2^; 95% CI, 0.209-0.427 vs 0.157 g/m^2^; 95% CI, 0.060-0.254.; ANCOVA *P* < .001) (Figure 1; eTables 3 and 4 in the Supplement). When dividing by gestational age categories, the association between systolic blood pressure and LV mass index in the adjusted regression models was significant across all groups but was greatest in those born very and extremely preterm (<32 weeks’ gestation), with a 0.394 g/m^2^ (95% CI, 0.213-0.574) greater LV mass index per 1–mm Hg elevation in systolic blood pressure compared with 0.250 g/m^2^ (95% CI, 0.113-0.387) in those born moderately preterm (32-36 weeks’ gestation) (ANCOVA *P* = .03) and 0.157 g/m^2^ (95% CI, 0.060-0.254) in those born at term (≥37 weeks’ gestation) (ANCOVA *P* < .001) (Figure 2 and Table 3). The adjusted regression lines were also significantly different between moderately preterm-born and term-born individuals (eTable 5 in the Supplement). Although there was a significant, positive association between systolic blood pressure and ejection fraction in the term-born adults (0.079%; 95% CI, 0.017-0.140 per 1–mm Hg elevation in systolic blood pressure), this association was not significant in preterm-born adults. In multivariable regression, there was a significant, positive association between systolic blood pressure and LV mass to end-diastolic volume ratio in the preterm-born adults (2.38 × 10^−3^ g/mL; 95% CI, 8.15 × 10^−4^ to 3.94 × 10^−3^ per 1–mm Hg elevation in systolic blood pressure) but not in term-born adults, with a significant difference between regression lines (ANCOVA *P* < .001). This association was greatest in adults born very and extremely preterm (3.56 × 10^−3^ g/mL; 95% CI, 1.01 × 10^−3^ to 6.11 × 10^−3^ per 1–mm Hg elevation in systolic blood pressure), with the regression line differing significantly for this group compared with moderately preterm (ANCOVA *P* < .001) and term-born adults (ANCOVA *P* < .001) (Table 3; eTables 4 and 5 in the Supplement). The regression results were not affected by additional adjustment for family history of hypertension (eResults in the Supplement). Subgroup analyses in male and female individuals and using 24-hour ambulatory and central systolic blood pressure measures also showed consistent findings (eFigures 2-5 in the Supplement).

**Table 3.** Multivariable Regression Coefficients for Systolic Blood Pressure in Multiple Regression Models of Key Left Ventricular Parameters Within Gestational Age Group Categories^a^

CMR parameters	Model	Very and extremely preterm-born adults (<32 wk gestation)				Moderately preterm-born adults (32-36 wk gestation)				Term-born adults (≥37 wk gestation)			
		B (per 1–mm Hg SBP)	95% CI		*P* value	B (per 1–mm Hg SBP)	95% CI		*P* value	B (per 1–mm Hg SBP)	95% CI		*P* value
			Lower bound	Upper bound			Lower bound	Upper bound			Lower bound	Upper bound	
Myocardium Mass/BSA, g/m^2^	Unadjusted	0.475	0.297	0.654	<.001	0.281	0.135	0.427	<.001	0.288	0.189	0.387	<.001
	Adjusted	0.394	0.213	0.574	<.001	0.250	0.113	0.387	<.001	0.157	0.060	0.254	.002
Ejection fraction, %	Unadjusted	0.068	−0.036	0.171	.20	−0.010	−0.102	0.082	.84	0.066	0.010	0.123	.02
	Adjusted	0.100	−0.015	0.216	.09	−0.031	−0.129	0.067	.53	0.079	0.017	0.140	.01
ED volume/BSA, mL/m^2^	Unadjusted	0.170	0.010	0.330	.04	0.192	−0.001	0.386	.05	0.139	0.005	0.273	.04
	Adjusted	0.157	−0.016	0.329	.07	0.179	−0.011	0.393	.07	0.092	−0.043	0.228	.18
Mass/ED volume, g/mL	Unadjusted	4.47 × 10^−3^	2.16 × 10^−3^	6.78 × 10^−3^	<.001	1.53 × 10^−3^	−5.40 × 10^−5^	3.12 × 10^−3^	.06	2.44 × 10^−3^	1.22 × 10^−3^	3.67 × 10^−3^	<.001
	Adjusted	3.56 × 10^−3^	1.01 × 10^−3^	6.11 × 10^−3^	.007	1.04 × 10^−3^	−6.83 × 10^−4^	2.74 × 10^−3^	.24	1.08 × 10^−3^	−2.31 × 10^−4^	2.40 × 10^−3^	.11

Abbreviations: B, unstandardized coefficients with 95% confidence intervals; BSA, body surface area; CMR, cardiac magnetic resonance; ED, end-diastole; SBP, systolic blood pressure.

^a^ The regression coefficients for systolic blood pressure with the individual CMR parameters (dependent variables) are shown for both unadjusted (bivariate) and adjusted (multivariable) regression models. In multivariable models, independent variables were systolic blood pressure, age, sex, birth weight *z* score, and body mass index (multivariable regression coefficients shown for systolic blood pressure).

## Discussion

In a large cohort of young adults, we have investigated the association between systolic blood pressure elevation and LV structure and function in preterm-born individuals using CMR. In regression analyses of systolic blood pressure and LV mass index and LV mass to end-diastolic volume ratio, there was a leftward shift in the slopes in the young adults born preterm compared with those born at term. In addition, there was a 2.5-fold greater LV mass index per 1–mm Hg systolic blood pressure elevation in very and extremely preterm-born young adults and a 1.6-fold greater LV mass index per 1–mm Hg systolic blood pressure elevation in moderately preterm-born young adults compared with term-born young adults. Furthermore, LV mass to end-diastolic volume ratio per 1–mm Hg systolic blood pressure elevation in the very and extremely preterm-born young adults was 3.4-fold greater compared with those born moderately preterm and 3.3-fold greater compared with those born at term, with no difference between the latter 2 groups.

As the immature heart transitions to the ex utero environment, it must undergo cellular, structural, and functional adaptations to deal with the dramatic shift in pressure and oxygen.^16^ In sheep born preterm, this early transition leads to a disruption in normal postnatal myocardial maturation that may increase later cardiac susceptibility to failure.^17^ Hearts from piglets delivered preterm were unable to maintain LV cardiac output when challenged ex vivo with an increased afterload.^18^ Similarly, in a rat model of preterm birth conditions, there was accelerated myocardial hypertrophy, diffuse fibrosis, and impaired systolic function in the LV prior to systemic blood pressure elevation.^5^ As a result of their reduced LV functional reserve, they were more likely to go into heart failure when hemodynamically challenged with angiotensin II infusion, although angiotensin receptor blocker treatment prevented the development of cardiac alterations.^4^ Human studies from birth through to young adulthood have now demonstrated that LV function and structure are altered in individuals born preterm,^3^ including an inability to meet the demands of physiologic stress in adolescence and young adulthood.^6,7,19,20^ The lower myocardial functional reserve seen in preterm-born young adults in response to acute stress may in part explain why the risk of early heart failure is so much greater in this population.^1,21^

Given the underlying morphologic impairments in the preterm heart, it is conceivable that blood pressure elevation would have a greater effect on cardiac remodeling in this population. Hypertension is known to cause LV structural and functional adaptations, including wall thickening and impaired systolic and diastolic function, especially with more advanced disease in older populations.^22,23,24^ Our study adds to the limited cardiac imaging data available in young adults with mild to moderate hypertension using CMR, which is widely considered the criterion standard modality for assessing LV structure and function.^25^ While most LV parameters did not differ significantly between term-born young adults with and without hypertension, LV ejection fraction was higher in the hypertensive group, with a significant, positive association between LV ejection fraction and systolic blood pressure that was not seen in those born preterm. Higher midventricular peak systolic circumferential strain accompanied the higher LV ejection fraction in the term group, which could indicate systolic hypercontractility to compensate for increased afterload. This was not observed in the preterm-born young adults, which may suggest an inability to adapt to early stages of blood pressure elevation. Interestingly, in the preterm-born young adults, we did not see the extent of LV structural or functional changes we might have expected when comparing individuals with and without hypertension. Nevertheless, when quantifying the continuous association between LV parameters and increasing systolic blood pressure, there was a steeper slope in the adjusted regression line between both LV mass index and LV mass to end-diastolic volume ratio with systolic blood pressure in the preterm group that increased with the degree of prematurity. Furthermore, the leftward shift in the preterm regression lines meant that both LV mass index and LV mass to end-diastolic volume ratio were greater for any given systolic blood pressure. This suggests that blood pressure alone is not the driving factor for the observed LV differences between preterm-born and term-born young adults and that there are unique drivers of increased cardiovascular risk in the preterm population.^26^

### Limitations

A limitation of the study was that the main analyses were based on single-arm clinic blood pressure measurements. Despite this, we were able to perform subgroup analyses in approximately 75% of the cohort using 24-hour and surrogate central blood pressure measures, which showed consistent results. Future studies to more accurately quantify the effect of pulse pressure amplification are needed, using methods such as CMR aortic distension waveforms to assess central systolic blood pressure.^27^ Although we were able to identify that the greatest increase in LV mass index and LV mass to end-diastolic volume ratio in association with increasing systolic blood pressure was in those born at less than 32 weeks’ gestation, our sample size of individuals born at less than 28 weeks’ gestation (extremely preterm) was too small to investigate in isolation. In a study by Goss et al,^28^ it was shown that extremely preterm-born adolescents and young adults had smaller LV volumes and mass, as well as a more hypercontractile LV compared with their term-born peers. However, all participants in that study were normotensive. Given that this more premature group is at an exponentially greater risk of early heart failure,^1^ further work is needed to understand cardiac remodeling in response to hypertension in the most premature individuals. Owing to retrospective perinatal data collection in a significant proportion of our cohort, we did not have complete information on all perinatal complications, such as infection rates, days of ventilation, and hypertensive pregnancies. Further studies will be needed to explore whether these factors affect the findings. Nevertheless, our complete gestational age and birth weight data allowed us to determine that the degree of prematurity may be important to how the preterm heart responds to blood pressure elevation and could add value to young adult risk stratification. While our study sample size of individuals with stage II hypertension was limited, because most young adults in this blood pressure range would be taking medication, the leftward shift in the association between LV changes and systolic blood pressure suggests that, even in the early stages of blood pressure elevation, appropriate clinical screening and monitoring may be warranted. Further research is needed to investigate this and to exclude the possibility of coassociation between LV changes and higher systolic blood pressure.

## Conclusions

In summary, young adults born preterm have an altered cardiac structure and function compared with their term-born peers, with greater remodeling in response to systolic blood pressure elevation. Further research should include serial study visits with cardiac imaging in the same individuals over time to better understand how the heart remodels in response to blood pressure elevation and which factors may increase or decrease risk to guide potential interventions. Given that preterm birth affects more than 10% of live births and that survival of even the most premature individuals continues to rise, lifelong clinical follow-up may be needed to prevent the emergence of early cardiovascular disease in this population.

## Notes

Supplement. eMethods. eResults. **eFigure.** Flow of information through the different phases of the review **eTable 1. **Baseline participant characteristics **eTable 2. **Family medical history divided by birth history and blood pressure subgroups **eTable 3.** Multivariable regression coefficients for systolic blood pressure in multiple regression models of key left ventricular parameters **eTable 4.** Systolic blood pressure versus left ventricular parameter regression line comparisons between preterm-born and term-born young adults **eTable 5. **Systolic blood pressure versus left ventricular parameter regression line comparisons between gestational age group categories **eFigure 1.** Radar plot showing the change in left ventricular parameters across blood pressure and birth history subgroups **eFigure 2.** Relationship between systolic blood pressure and LV mass index in preterm-born and term-born female adults **eFigure 3.** Relationship between systolic blood pressure and LV mass index in preterm-born and term-born male adults **eFigure 4.** Relationship between central systolic blood pressure and LV mass index in preterm-born and term-born adults **eFigure 5.** Relationship between 24-hour ambulatory systolic blood pressure and LV mass index in preterm-born and term-born adults
