# Supplementary material for: Association of Systolic Blood Pressure Elevation With Disproportionate Left Ventricular Remodeling in Very Preterm-Born Young Adults: The Preterm Heart and Elevated Blood Pressure
Source: JAMA Cardiol. 2021 May 12;6(7):821–9. doi: 10.1001/jamacardio.2021.0961 (PMC8117059; doi:10.1001/jamacardio.2021.0961)
Supplement: Supplement. — eMethods. eResults. eFigure. Flow of information through the different phases of the review eTable 1. Baseline participant characteristics eTable 2. Family medical history divided by birth history and blood pressure subgroups eTable 3. Multivariable regression coefficients for systolic blood pressure in multiple regression models of key left ventricular parameters eTable 4. Systolic blood pressure versus left ventricular parameter regression line comparisons between preterm-born and term-born young adults eTable 5. Systolic blood pressure versus left ventricular parameter regression line comparisons between gestational age group categories eFigure 1. Radar plot showing the change in left ventricular parameters across blood pressure and birth history subgroups eFigure 2. Relationship between systolic blood pressure and LV mass index in preterm-born and term-born female adults eFigure 3. Relationship between systolic blood pressure and LV mass index in preterm-born and term-born male adults eFigure 4. Relationship between central systolic blood pressure and LV mass index in preterm-born and term-born adults eFigure 5. Relationship between 24-hour ambulatory systolic blood pressure and LV mass index in preterm-born and term-born adults [file jamacardiol-e210961-s001.pdf]

Supplemental Online Content

Mohamed A, Marciniak M, Williamson W, et al. Association of systolic blood pressure elevation with disproportionate left ventricular remodeling in very preterm-born young adults. *JAMA Cardiol*. Published online May 12, 2021. doi:10.1001/jamacardio.2021.0961

eMethods.

eResults.

eTable 1. Baseline participant characteristics

eTable 2. Family medical history divided by birth history and blood pressure subgroups

eTable 3. Multivariable regression coefficients for systolic blood pressure in multiple regression models of key left ventricular parameters

eTable 4. Systolic blood pressure versus left ventricular parameter regression line comparisons between preterm-born and term-born young adults

eTable 5. Systolic blood pressure versus left ventricular parameter regression line comparisons between gestational age group categories

eFigure 1. Radar plot showing the change in left ventricular parameters across blood pressure and birth history subgroups

eFigure 2. Relationship between systolic blood pressure and LV mass index in preterm-born and term-born female adults

eFigure 3. Relationship between systolic blood pressure and LV mass index in preterm-born and term-born male adults

eFigure 4. Relationship between central systolic blood pressure and LV mass index in preterm-born and term-born adults

eFigure 5. Relationship between 24-hour ambulatory systolic blood pressure and LV mass index in preterm-born and term-born adults

This supplemental material has been provided by the authors to give readers additional information about their work.

© 2021 Mohamed A et al. *JAMA Cardiology.*

SUPPLEMENTAL METHODS

24-Hour ambulatory blood pressure measurements

Twenty-four-hour ambulatory blood pressure monitoring was initiated at the end of the study visits, on the same day as the cardiovascular magnetic resonance (CMR) scans, using oscillometric, ambulatory devices (TM-2430, A&D Instruments, Abingdon, UK). Correct cuff size was chosen based on arm circumference. Subjects were instructed to remain still during measurements. Measurements were automatically taken every 30 minutes during daytime (from 7:00 am to 11:00pm) and then hourly during night-time (from 11:00 pm to 7:00 am). Subjects completed a diary documenting the timing of their sleep to allow accurate discrimination of awake and sleep periods. Normal daily activities were encouraged, with participants asked to keep their left arm relaxed and still when measurements were taking place. Data analysis was done using ABPM Data Analysis Software for Windows (version 2.40;A&D Instruments, Abingdon, UK). Data were available for 339 out of the 468 individuals in the cohort as these measurements were included in the protocol through an ethical amendment after the study had started.

Central blood pressure measurements

The Vicorder system (Skidmore Medical, Bristol, UK), a cuff-based device around the upper arm that derives central (aortic) blood pressure from brachial blood pressure waveforms, was measured on the same day as the CMR scans. Participants were seated for at least five minutes before three resting brachial blood pressure readings were taken on the left arm with a one-minute interval in between. The final two readings were averaged for subsequent analyses of central blood pressure. To derive these measures, the Vicorder cuff was statically inflated at the level of 70mmHg using a volume-displacement technique. A transfer function to the brachial waveform was then applied by the Vicorder software to derive the central blood pressure reading.[1] Data were available for 352 out of the 468 individuals in the cohort as these measurements were included in the protocol through an ethical amendment after the study had started.

© 2021 Mohamed A et al. *JAMA Cardiology.*

SUPPLEMENTAL RESULTS

Impact of family history of hypertension

A family history of hypertension was more prevalent in hypertensive preterm-born adults than in hypertensive term-born adults (Supplemental eTable 2). We have therefore explored whether additional adjustment for family history of hypertension had an impact on the relationship between systolic blood pressure and left ventricular (LV) mass index. When dividing based on birth history, there was a stronger relationship between systolic blood pressure and LV mass index in the preterm- compared to term-born young adults (R^2=14.7%, P<.001 and R^2=3.68%, P=.002) when adjusting for age, sex, birthweight z-score, body mass index and family history of hypertension. This was also indicated by the greater slope in the preterm- compared to term-born young adults (0.321 vs 0.155g/m^2 per 1mmHg elevation in systolic blood pressure) (ANCOVA P<.001). When dividing by gestational age category, the slope and R^2 increased in a similar manner with the degree of prematurity when adjusting for age, sex, birthweight z-score, body mass index and family history of hypertension. The greatest slope was in the extremely and very preterm-born adults (0.396g/m^2 per 1mmHg increase in systolic blood pressure, R^2=17.1%, P<.001) compared to both moderately preterm (0.256g/m^2 per 1mmHg increase in systolic blood pressure, R^2=12.8%, P<.001; ANCOVA P=.03) and term-born adults (0.155g/m^2 per 1mmHg increase in systolic blood pressure, R^2=3.68%, P=0.002; ANCOVA P<.001).

The relationship between systolic blood pressure and LV mass index to end-diastolic volume ratio when dividing based on birth history was significant in the preterm-born adults but not those born at term (R^2=6.45%, P=.003 and R^2=1.00%, P=.11) when adjusting for age, sex, birthweight z-score, body mass index and family history of hypertension. This was also indicated by the greater slope in the preterm- compared to term-born young adults ($2.40x10^{-3}$ vs $1.08x10^{-3}$ g/mL per 1mmHg elevation in systolic blood pressure) (ANCOVA P<.001). When dividing by gestational age category, the slope and R^2 increased in a similar manner with the degree of prematurity when adjusting for age, sex, birthweight z-score, body mass index and family history of hypertension. The slope and R^2 were only significant in the extremely and very preterm-born adults ($3.41x10^{-3}$ g/mL per 1mmHg increase in systolic blood pressure, R^2=13.8%, P=.009) and differed significantly compared to both moderately preterm ($1.05x10^{-3}$ g/mL per 1mmHg increase in systolic blood pressure, R^2=1.45%, P=.22; ANCOVA P<.001) and term-born adults ($1.08x10^{-3}$ g/mL per 1mmHg increase in systolic blood pressure, R^2=1.00%, P=.11; ANCOVA P<.001).

© 2021 Mohamed A et al. *JAMA Cardiology.*

Supplemental eTable 1: Baseline Participant Characteristics

	Preterm-Born Adults (n=200)	Term-Born Adults (n=268)	P value
**Demographics and Anthropometrics**			
Age (years)	25.7 ± 3.94	26.5 ± 4.59	.05
Male, n (%)	91 (45.5)	136 (50.7)	.31
Height (cm)	170 ± 10.11	174 ± 9.14	<.001
Weight (kg)	69.2 ± 13.46	72.4 ± 13.02	.04
BMI (kg/m^2)	24.0 ± 3.73	23.9 ± 3.41	.48
Birth Weight (grams)	1628 ± 631	3445 ± 424	<.001
Birth Weight (z-score)	-0.26 ± 1.05	0.09 ± 0.89	<.001
Small for Gestational Age, n (%)	10 (5.0)	5 (1.9)	.99
Gestational Age (weeks)	31.3 ± 2.98	39.6 ± 1.14	<.001
$< 28^{+0}$ weeks, n (%)	21 (10.5)	-	-
$28^{+0} – 31^{+6}$ weeks, n (%)	74 (37.0)	-	-
$32^{+0} – 36^{+6}$ weeks, n (%)	105 (52.5)	-	-
**Family medical history of, n (%)**			
Ischemic Heart Disease	17 (8.5)	13 (4.9)	.09
Hypertension	70 (35.0)	56 (20.9)	.001
Stroke	13 (6.5)	4 (1.5)	.002
Diabetes Mellitus	19 (9.5)	21 (7.8)	.44
High Cholesterol	43 (21.5)	48 (17.9)	.36
**Biochemistry**			
Total Cholesterol (mmol/L)	4.66 ± 1.04	4.46 ± 0.95	.02
HDL (mmol/L)	1.49 ± 0.37	1.44 ± 0.35	.27
LDL (mmol/L)	2.78 ± 0.79	2.59 ± 0.79	.003
Triglycerides (mmol/L)	1.23 ± 0.97	0.99 ± 0.59	<.001
High Sensitivity CRP (mg/L)	1.92 ± 3.54	1.59 ± 3.21	.31
Glucose (mmol/L)	4.96 ± 0.43	4.82 ± 0.47	<.001
Insulin (pmol/L)	53.6 ± 30.74	47.17 ± 45.16	.06
Homa-B (%)	98.0 ± 33.90	93.5 ± 32.68	.27
Homa-S (%)	124.0 ± 53.05	156.3 ± 72.17	<.001
Homa-IR	0.84 ± 0.33	0.76 ± 0.38	.03
**Brachial Blood Pressure (mm Hg)**			
Resting Systolic	121.0 ± 11.5	117.9 ± 11.2	.001
Resting Diastolic	72.9 ± 8.1	71.8 ± 8.7	.06

Group characteristics presented as mean ± SD, n (%), or range. *P* values adjusted for sex and age. BMI indicates body mass index; CRP, C-reactive protein; HDL, high density lipoprotein; HT, hypertensive; LDL, low density lipoprotein; NT, normotensive.

© 2021 Mohamed A et al. *JAMA Cardiology.*

Supplemental eTable 2: Family Medical History Divided by Birth History and Blood Pressure Subgroups

	Preterm-Born Adults		P[a]	Term-Born Adults		P[b]	P[c]	P[d]	P[e]
	Normotensive (n=139)	Hypertensive (n=61)		Normotensive (n=205)	Hypertensive (n=63)				
Ischemic Heart Disease, n (%)	10 (7.2)	7 (11.5)	.30	10 (4.9)	3 (4.8)	.93	.33	.46	.18
Hypertension, n (%)	42 (30.2)	28 (45.9)	.04	43 (21.0)	13 (20.6)	.92	.05	.26	.007
Stroke, n (%)	8 (5.8)	5 (8.2)	.71	1 (0.5)	3 (4.8)	.02	.001	.51	.48
Diabetes Mellitus, n (%)	10 (7.2)	9 (14.8)	.16	17 (8.3)	4 (6.3)	.78	.74	.43	.08
High Cholesterol, n (%)	27 (19.4)	16 (26.2)	.35	38 (18.5)	10 (15.9)	.86	.88	.65	.21

P values adjusted for age and sex.

P[a] preterm-born normotensive vs preterm-born hypertensive
P[b] term-born normotensive vs term-born hypertensive
P[c] preterm-born normotensive vs term-born normotensive
P[d] preterm-born normotensive vs term-born hypertensive
P[e] preterm-born hypertensive vs term-born hypertensive

© 2021 Mohamed A et al. *JAMA Cardiology*.

Supplemental eTable 3: Multivariable Regression Coefficients for Systolic Blood Pressure in Multiple Regression Models of Key Left Ventricular Parameters

CMR Parameters	Model	Preterm-born Young Adults				Term-born Young Adults			
			95% confidence intervals				95% confidence intervals		
		B (per 1mmHg SBP)	Lower Bound	Upper Bound	P value	B (per 1mmHg SBP	Lower Bound	Upper Bound	P value
**Myocardium Mass/BSA (g/m²)**	Unadjusted	0.363	0.247	0.478	<.001	0.288	0.189	0.387	<.001
	**Adjusted**	0.318	0.209	0.427	<.001	0.157	0.060	0.254	.002
**Ejection Fraction (%)**	Unadjusted	0.024	-0.044	-0.092	.49	0.066	0.010	0.123	.02
	**Adjusted**	0.024	-0.049	0.097	.52	0.079	0.017	0.140	.01
**ED Volume/BSA (mL/m²)**	Unadjusted	0.188	0.060	0.315	.004	0.139	0.005	0.273	.04
	**Adjusted**	0.165	0.034	0.296	.05	0.092	-0.043	0.228	.18
**Mass/ED Volume (g/mL)**	Unadjusted	$2.72x10^{-3}$	1.2710^{-3}	4.1710^{-3}	<.001	$2.45x10^{-3}$	1.2210^{-3}	3.6710^{-3}	<.001
	**Adjusted**	$2.38x10^{-3}$	$8.15x10^{-4}$	$3.94x10^{-3}$	.003	$1.08x10^{-3}$	$-2.31x10^{-4}$	$2.40x10^{-3}$	.11

B indicates unstandardised coefficients with 95% confidence intervals; BSA, body surface area; CMR, cardiac magnetic resonance; ED, end-diastole; SBP, systolic blood pressure. The regression coefficients for systolic blood pressure with the individual CMR parameters (dependent variables) are shown for both unadjusted (bivariate) and adjusted (multivariable) regression models. In multivariable models, independent variables were systolic blood pressure, age, sex, birth weight z-score, and body mass index (multivariable regression coefficients shown for systolic blood pressure).

© 2021 Mohamed A et al. *JAMA Cardiology.*

Supplemental eTable 4: Systolic Blood Pressure versus Left Ventricular Parameter Regression Line Comparisons between Preterm-Born and Term-Born Young Adults

CMR Parameters	Model	Sum square	F	P value
**Myocardium Mass/BSA (g/m²)**	Unadjusted	7080.0	81.6	<.001
	**Adjusted**	8141.8	122.0	<.001
**Mass/ED volume (g/mL)**	Unadjusted	4.018	299.4	<.001
	**Adjusted**	4.041	310.7	<.001

BSA indicates body surface area; ED, end-diastole. In multivariable models, independent variables were systolic blood pressure, age, sex, birth weight z-score, and body mass index.

© 2021 Mohamed A et al. *JAMA Cardiology.*

Supplemental eTable 5: Systolic Blood Pressure versus Left Ventricular Parameter Regression Line Comparisons between Gestational Age Group Categories

CMR Parameters	Model	Term vs moderately preterm ($>37^{+0/7}$ weeks vs $32^{+0/7} – 36^{+6/7}$ weeks)			Term vs very and extremely preterm ($>37^{+0/7}$ weeks vs $<32^{+0/7}$ weeks)			Very and extremely preterm vs moderately preterm ($<32^{+0/7}$ weeks vs $32^{+0/7} – 36^{+6/7}$ weeks)		
		**Sum square**	**F**	**P value**	**Sum square**	**F**	**P value**	**Sum square**	**F**	**P value**
**Myocardium Mass/BSA (g/m²)**	Unadjusted	3184.7	38.72	<.001	6283.0	70.86	<.001	466.6	5.322	.02
	**Adjusted**	4290.9	68.55	<.001	6808.1	98.08	<.001	320.2	4.848	.03
**Mass/ED volume (g/mL)**	Unadjusted	1.711	144.03	<.001	3.661	266.09	<.001	0.310	24.49	<.001
	**Adjusted**	1.816	162.39	<.001	3.650	272.75	<.001	0.299	23.88	<.001

BSA indicates body surface area; ED, end-diastole. In multivariable models, independent variables were systolic blood pressure, age, sex, birth weight z-score, and body mass index.

© 2021 Mohamed A et al. *JAMA Cardiology.*

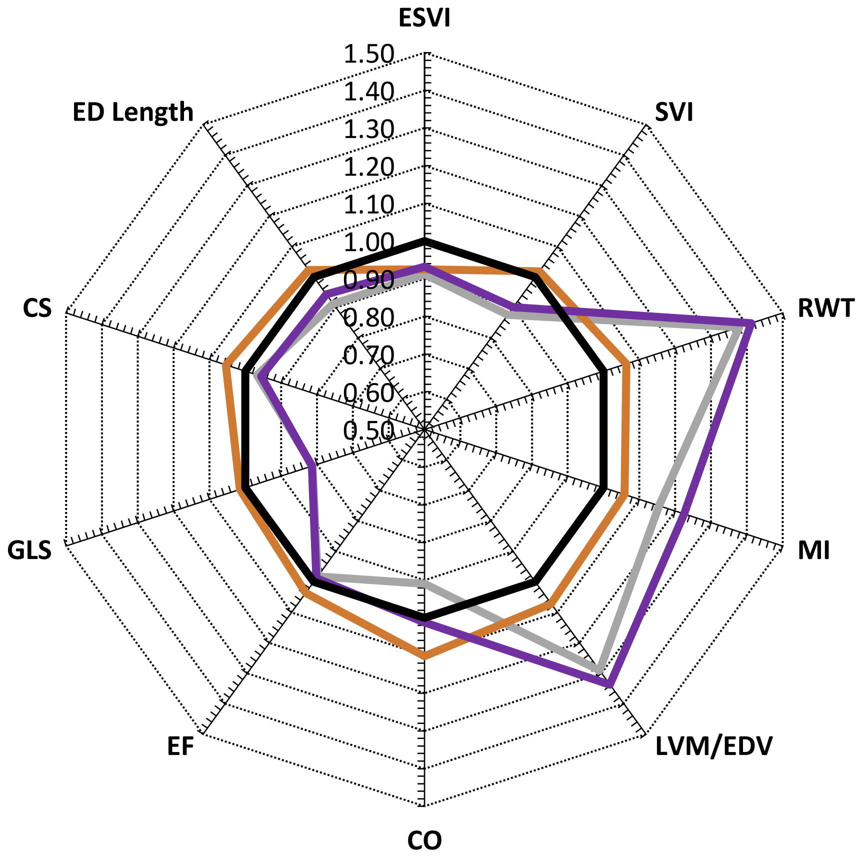

Supplemental eFigure 1 – Radar plot showing the change in left ventricular parameters across blood pressure and birth history subgroups. Key LV parameters are presented as ratios of the average values within the groups relative to the term-born normotensive reference group. The black reference line (at 1.00) represents the term-born normotensive individuals. Each parameter for the other groups is shown as a ratio, relative to term-born normotensive individuals. While differences in left ventricular parameters in the term-born hypertensive group (orange) were relatively small compared to the term-born normotensive group, both preterm-born normotensive (grey) and hypertensive (purple) individuals had significant left ventricular structural and functional changes. CO indicates cardiac output; CS, mid-ventricular peak systolic circumferential strain; ED length, end-diastolic length; EF, ejection fraction; ESVI, end-systolic volume index; GLS, global longitudinal peak systolic strain; LVM/EDV, left ventricular mass to end-diastolic volume ration; MI, mass index; RWT, relative wall thickness; SVI, stroke volume index.

© 2021 Mohamed A et al. *JAMA Cardiology.*

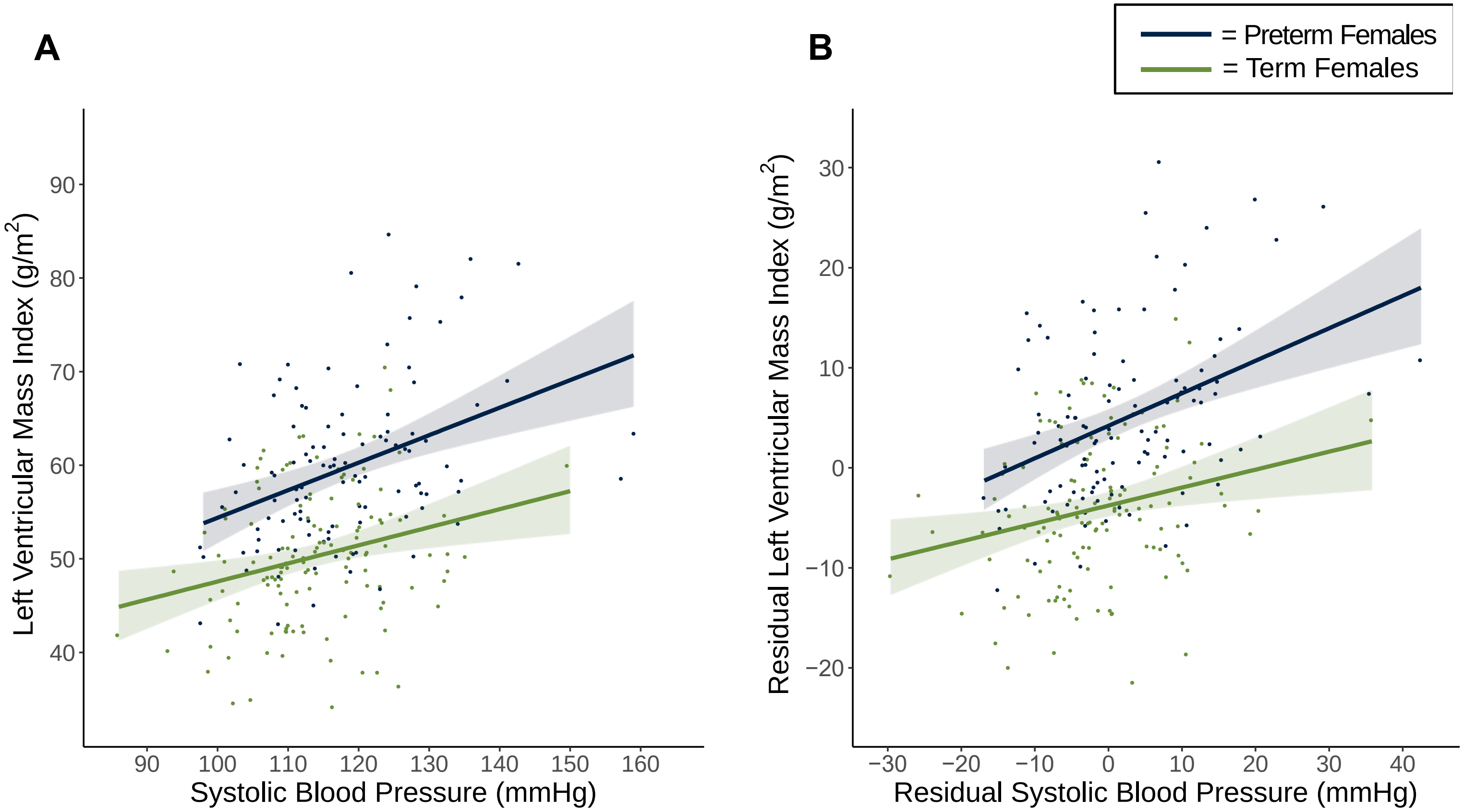

eFigure 2: Relationship between systolic blood pressure and LV mass index in preterm-born and term-born female adults. A, Scatterplot demonstrating the relationship between systolic blood pressure and LV mass index in preterm-born (blue) and term-born (green) young adult females. There was a stronger relationship in both preterm- and term-born females for systolic blood pressure vs LV mass index (R^2=15.0%, P<.001 and R^2=6.60%, P=.003). The slope was slightly greater for the preterm-born vs term-born female adults (0.294 vs 0.193g/m^2 per 1mmHg elevation in systolic blood pressure) and there was a leftward shift in the regression line in those born preterm compared to those born at term (ANCOVA P<.001). **B**, Partial regression plot demonstrating the relationship between systolic blood pressure residuals and LV mass index residuals in preterm- and term-born young adult females, with adjustment for age, birthweight z-score and body mass index. There was a stronger relationship between systolic blood pressure and LV mass index in the preterm-born compared to term-born young adult females (R^2=16.4%, P<.001 and R^2=6.20%, P=.005). This was also indicated by the greater slope in the preterm-born compared to term-born young adult females (0.322 vs 0.187g/m^2 per 1mmHg elevation in systolic blood pressure) (ANCOVA P<.001).

© 2021 Mohamed A et al. *JAMA Cardiology.*

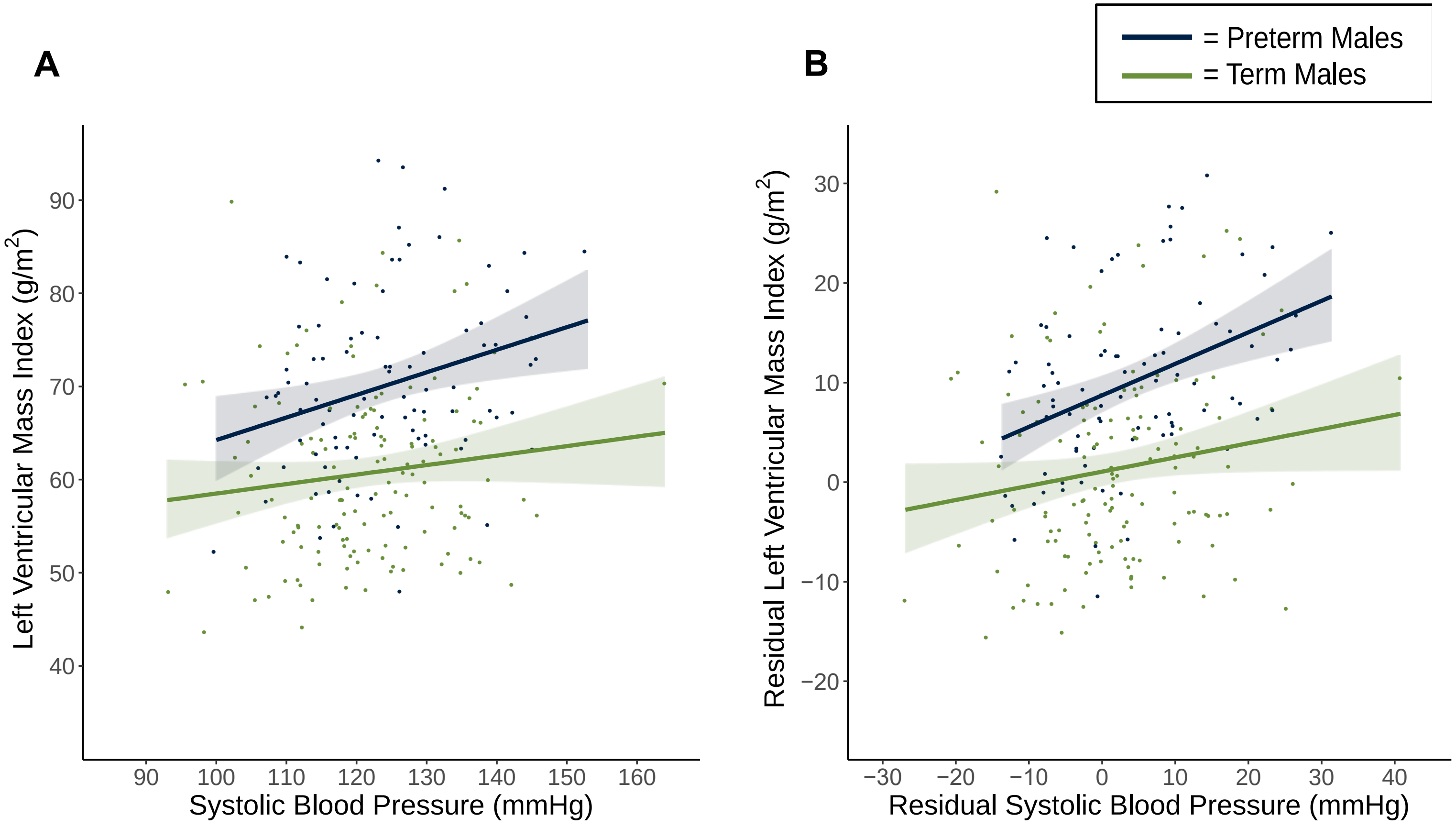

eFigure 3: Relationship between systolic blood pressure and LV mass index in preterm-born and term-born male adults. A, Scatterplot demonstrating the relationship between systolic blood pressure and LV mass index in preterm-born (blue) and term-born (green) young adult males. The relationship between systolic blood pressure and LV mass index was significant in the preterm-born males but not in the term-born males (R^2=12.6%, P=.005 and R^2=1.61%, P=.14). The slope was also greater for the preterm-born vs term-born male adults (0.252 vs 0.102g/m^2 per 1mmHg elevation in systolic blood pressure) and there was a leftward shift in the regression line in those born preterm compared to those born at term (ANCOVA P<.001). **B**, Partial regression plot demonstrating the relationship between systolic blood pressure residuals and LV mass index residuals in preterm-born and term-born young adult males, with adjustment for age, birthweight z-score and body mass index. There was a stronger relationship between systolic blood pressure and LV mass index in the preterm-born compared to term-born young adult males (R^2=14.1%, P=.001 and R^2=2.50%, P=.07) and greater slope in the preterm-born adult males (0.313 vs 0.133g/m^2 per 1mmHg elevation in systolic blood pressure) (ANCOVA P<.001).

© 2021 Mohamed A et al. *JAMA Cardiology*.

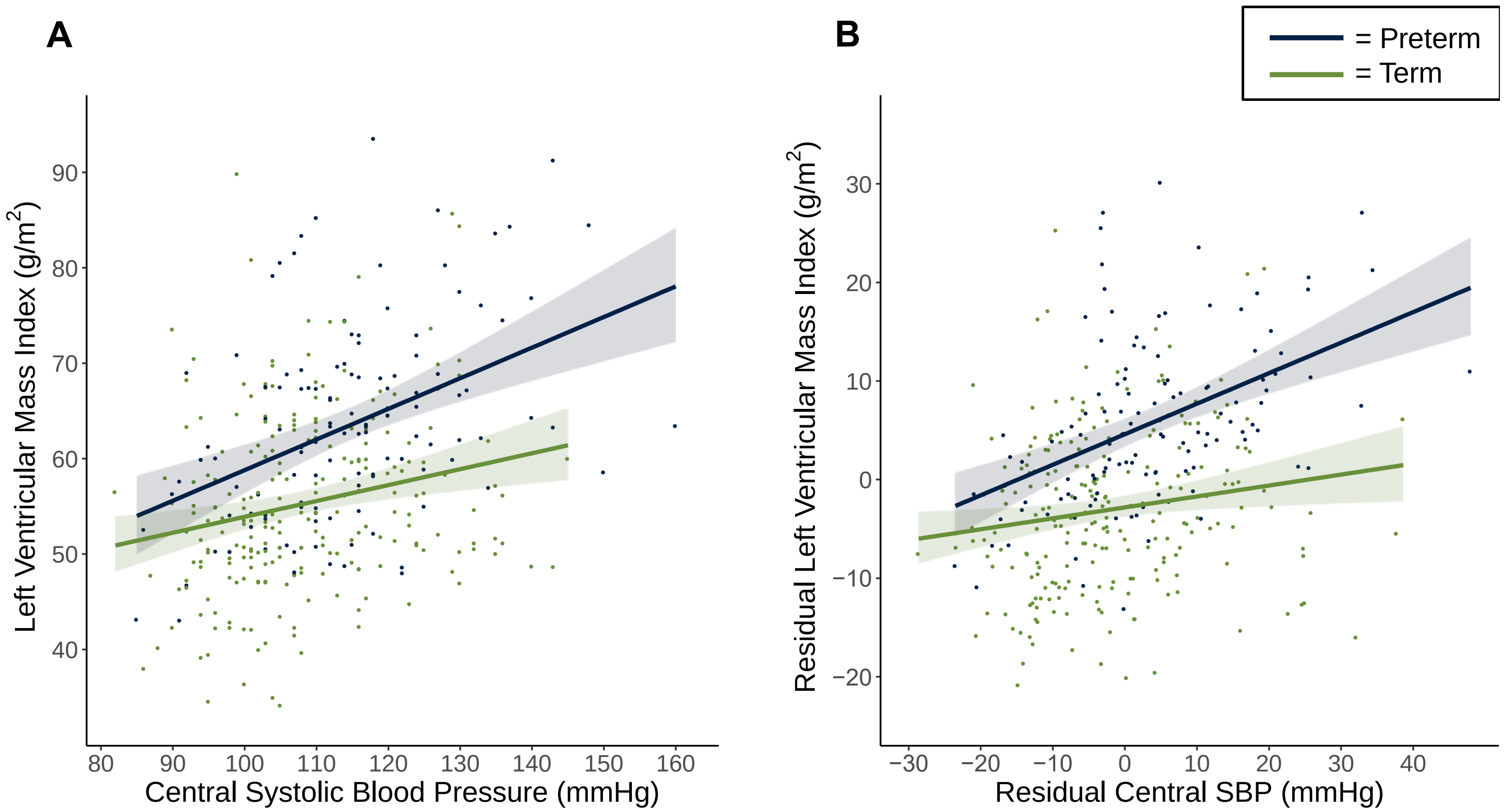

eFigure 4: Relationship between central systolic blood pressure and LV mass index in preterm-born and term-born adults. A, Scatterplot demonstrating the relationship between systolic blood pressure and LV mass index in preterm-born (blue) and term-born (green) young adults. There was a significant relationship in both preterm- and term-born young adults for central systolic blood pressure vs LV mass index (R^2=16.9%, P<.001 and R^2=4.58%, P=.001). The slope for this relationship was greater in the preterm- than term-born adults (0.312 vs 0.171g/m^2 per 1mmHg elevation in systolic blood pressure) (ANCOVA P<.001). **B**, Partial regression plot demonstrating the relationship between systolic blood pressure (SBP) residuals and LV mass index residuals in preterm-born and term-born young adults, with adjustment for age, sex, birthweight z-score and body mass index. There was a stronger relationship between central systolic blood pressure and LV mass index in the preterm- compared to term-born young adults (R^2=18.1%, P<.001 and R^2=2.16%, P=.03) and a greater slope in the preterm-born young adults (0.299 vs 0.103g/m^2 per 1mmHg elevation in systolic blood pressure) (ANCOVA P<.001).

© 2021 Mohamed A et al. *JAMA Cardiology*.

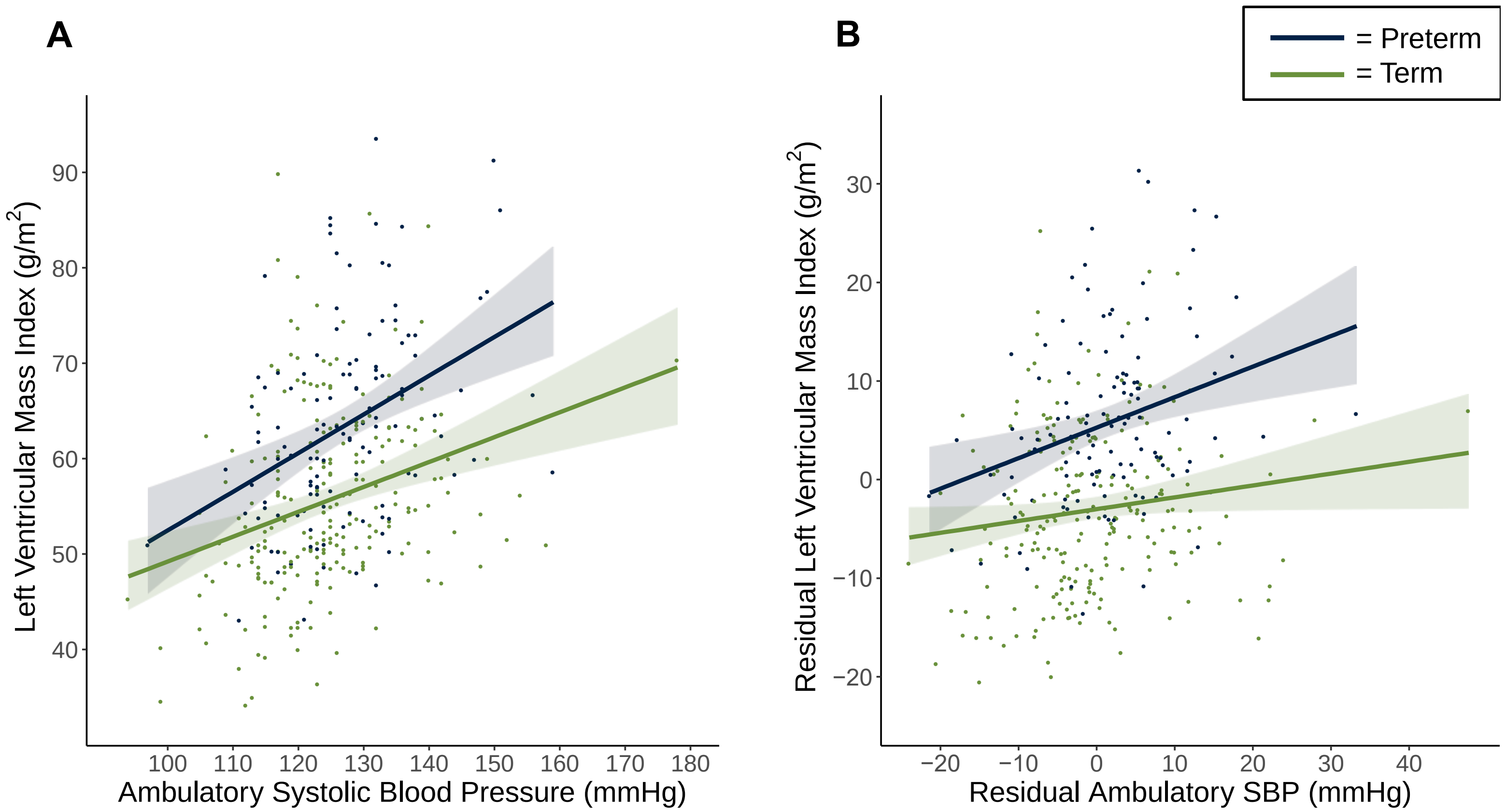

eFigure 5: Relationship between 24-hour ambulatory systolic blood pressure and LV mass index in preterm-born and term-born adults. **A,** Scatterplot demonstrating the relationship between systolic blood pressure and LV mass index in preterm-born (blue) and term-born (green) young adults. There was a significant relationship in both preterm- and term-born young adults for 24-hour systolic blood pressure vs LV mass index (R^2=14.6%, P<.001 and R^2=8.89%, P<.001). The slope for this relationship was greater in the preterm- than term-born adults (0.413 vs 0.283g/m^2 per 1mmHg elevation in systolic blood pressure) (ANCOVA P<.001). **B**, Partial regression plot demonstrating the relationship between systolic blood pressure (SBP) residuals and LV mass index residuals in preterm-born and term-born young adults, with adjustment for age, sex, birthweight z-score and body mass index. There was a stronger relationship for 24-hour systolic blood pressure (SBP) vs LV mass index in the preterm- compared to term-born young adults (R^2=10.1%, P=.001 and R^2=1.64%, P=.06), which was also indicated by the greater slope in the preterm-born adults (0.326 vs 0.113g/m^2 per 1mmHg elevation in systolic blood pressure) (ANCOVA P<.001).

© 2021 Mohamed A et al. *JAMA Cardiology.*

SUPPLEMENTAL REFERENCES

1. Pucci G, Cheriyan J, Hubsch A, et al. Evaluation of the Vicorder, a novel cuff-based device for the noninvasive estimation of central blood pressure. *Journal of hypertension.* 2013;31(1):77-85.

© 2021 Mohamed A et al. *JAMA Cardiology*.
